# Engineered Chimeric Peptides with IGF-1 and Titanium-Binding Functions to Enhance Osteogenic Differentiation In Vitro under T2DM Condition

**DOI:** 10.3390/ma15093134

**Published:** 2022-04-26

**Authors:** Jun-Jun Wang, Qian Xue, Ying-Jie Wang, Min Zhang, Yong-Jin Chen, Qian Zhang

**Affiliations:** State Key Laboratory of Military Stomatology, National Clinical Research Center for Oral Diseases, Shaanxi International Joint Research Center for Oral Diseases, Department of General Dentistry and Emergency, School of Stomatology, Air Force Medical University, Xi’an 710032, China; wl4999@126.com (J.-J.W.); l022295@163.com (Q.X.); wangyingj@fmmu.edu.cn (Y.-J.W.)

**Keywords:** implant, osseointegration, IGF-1, T2DM, peptide aptamer

## Abstract

Due to the complexity of the biomolecules and titanium (Ti) combination, it is a challenge to modify the implant surface with biological cytokines. The study proposed a new method for immobilizing cytokines on implant surface to solve the problem of low osseointegration under type 2 diabetes mellitus (T2DM) condition. This new modified protein that connected Ti-binding artificial aptamer minTBP-1 with Insulin-like growth factor I (IGF-I), had a special strong affinity with Ti and a therapeutic effect on diabetic bone loss. According to the copies of minTBP-1, three proteins were prepared, namely minTBP-1-IGF-1, 2minTBP-1-IGF-1 and 3minTBP-1-IGF-1. Compared with the other modified proteins, 3minTBP-1-IGF-1 adsorbed most on the Ti surface. Additionally, this biointerface demonstrated the most uniform state and the strongest hydrophilicity. In vitro results showed that the 3minTBP-1-IGF-1 significantly increased the adhesion, proliferation, and mineralization activity of osteoblasts under T2DM conditions when compared with the control group and the other modified IGF-1s groups. Real-time PCR assay results confirmed that 3minTBP-1-IGF-1 could effectively promote the expression of osteogenic genes, that is, ALP, BMP-2, OCN, OPG, and Runx2. All these data indicated that the 3minTBP-1-IGF-1 had the most efficacious effect in promoting osteoblasts osteogenesis in diabetic conditions, and may be a promising option for further clinical use.

## 1. Introduction

Currently, commercially pure titanium, known for its high degree of biocompatibility and good mechanical properties, remains the most widely used implant material in dental implant clinics [1]. The improvement of implant design, surface characteristics and surgical scheme makes the implant a safe and highly predictable procedure, with an average survival rate of 94.6% and an average success rate of 89.7% over 10 years [2]. The survival of implants initially depends on successful bone integration after implantation. However, in some pathological conditions, such as diabetes, osseointegration was impaired because of the poor bone quality [3]. A review of implant failure rate assessment through 13 studies showed that the highest failure rate of patients with diabetes was 14.3%, which was two times higher than that of normal patients [4]. According to the latest statistics from the International Diabetes Federation, the number of diabetic patients aged 20–79 is 424.9 million, which is three times the number of patients in 2000. By 2045, patients with diabetes may reach 629 million [3]. Among them, T2DM accounts for about 90% of DM [3]. Recently, the 40-year trend of tooth loss in diabetes and non-diabetic patients over the age of 25 has been investigated in the United States, which found that the number of missing teeth in diabetic patients is almost two times that of non-diabetic patients [5]. Therefore, it is of great significance to improve the success rate of implant-supported dentures in T2DM patients. The prominent features of T2DM are hyperglycemia, glucose intolerance, and insulin resistance. Some authors have suggested that T2DM could adversely interfere with the process of implant osseointegration, and incomplete and delayed bony formation around the implant leads to its ultimate failure [6,7]. It has been proven that the apoptosis rate of osteoblasts increases significantly and the mineralization rate decrease remarkably, which suggests that high glucose can impair the osteogenic function of osteoblasts [8]. Despite the extensive use of Ti implants and a substantially growing body of studies on the modification of implant surfaces, osseointegration of implants in a medically compromised condition such as T2DM still remains a challenge [2,9].

In humans, IGF-1 and its binding proteins have positive roles in the acquisition of peak bone mass (PBM) and the maintenance of bone mineral density (BMD) [10]. The anabolic hormone IGF-1 primarily regulates the linear and microarchitectural bone growth through osteoblast cells [11]. Clinically, the serum IGF-1 level in diabetic patients is significantly decreased [12]. Systemic treatment with IGF-1 reversed impaired bony healing in diabetic animals, possibly through improvement in the formation of bone and inhibition of resorption [13]. There is a continuing search for local IGF-1 delivery systems to improve the osseointegration of the implant. Some studies have investigated the use of co-polylactic/glycolic acid (PLGA, a biodegradable injectable polymer) microcapsules to make a preparation that releases IGF-1 over a certain period. However, how to load these microcapsules on the implant surface is still a problem [14,15].

Efficient immobilization of biomacromolecules on the mental surface is a crux to development in areas of regenerative medicine and tissue engineering [16]. To biologically modify a metal surface, the first step is typically the formation of an organic layer on the metal surface to introduce functional groups for binding to biological molecules [17]. The silane-coupling method, electrodeposition, photoimmobilization, and chemical treatment with molecules containing phosphate groups or catechol-related groups (dopamine or polydopamine) have recently been developed [18,19]. Because there are many possible residues participating in the conjugation reactions, proteins are often denatured due to the collapse of their tertiary structure [20]. These methods are sometimes not suitable for protein immobilization [20]. In the last several years, peptide aptamers (=binders) that specifically bind to the surfaces of various inorganic materials have been attracting attention in the field of bionanotechnology [20]. At present, many peptide aptamers that bind to a variety of inorganic materials have been screened out. Notably, a linear 6-mer new peptide aptamer, named minTBP-1 (RKLPDA), has been proved to have a specifically high affinity with Ti [21]. Considering its specific and strong binding ability, minTBP-1 could be used as a bridge between Ti and IGF-1 to form a new kind of modified protein, which endowed the chimeric peptide with a specific binding ability with Ti and the biological function of IGF-1. Due to the high molecular weight of IGF-1, only three chimeric peptides were produced, namely minTBP-1-IGF-1, 2minTBP-1-IGF-1, and 3minTBP-1-IGF-1. Whether these three modified proteins can effectively play the function of IGF-1, and promote the proliferation and differentiation of osteoblasts under T2DM conditions in vitro would be explored in subsequent experiments.

To the best of our knowledge, studies of endosseous dental implant placement in combination with the modified IGF-1s in diabetic conditions have not yet been performed. The (n)MinTBP-1-IGF-1-Ti complex represents a novel biomaterials-based tissue engineering strategy in bone regeneration. The purpose of this experiment is to verify the effect of functionalized (n)minTBP-1-IGF-1s modified titanium on its adhesion, proliferation, and differentiation of osteoblasts under T2DM conditions in vitro.

## 2. Materials and Methods

**Figure 1:**
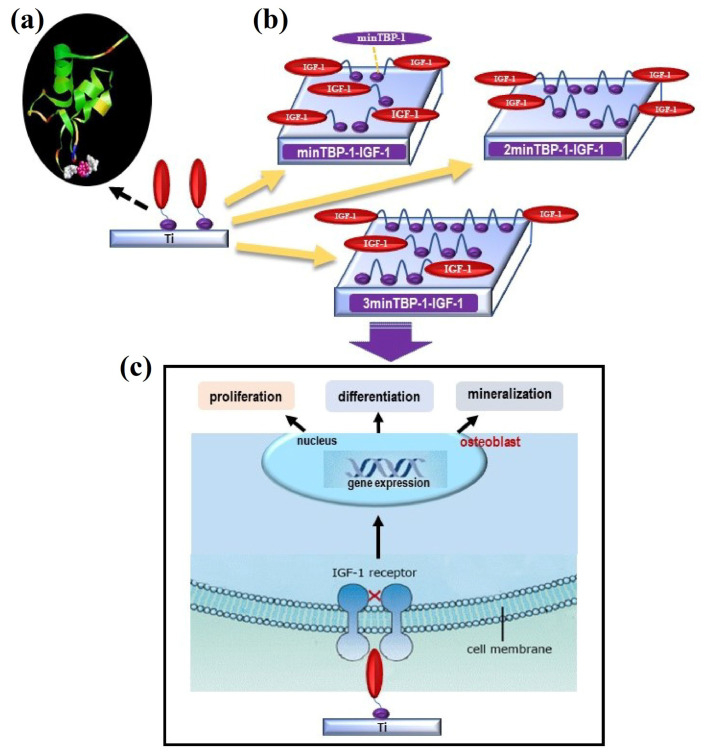
Preparation and experimental process of (n)minTBP-1-IGF-1-Ti complex: (**a**) conformational space of (n)minTBP-1-IGF-1; (**b**) samples preparation and (n)minTBP-1-IGF-1 loaded on Ti surfaces under T2DM condition; (**c**) the effects of functionalized Ti on osteogenesis under T2DM condition.

Figure 1 shows the biointerface preparation and the experimental process. MinTBP-1-IGF-1, 2minTBP-1-IGF-1, and 3minTBP-1-IGF-1 were loaded on Ti surfaces, and in vitro osteoblasts responses in terms of cell adhesion, proliferation, mineralization, and osteogenic gene expression are determined and discussed under diabetic condition.

### 2.1. Coating Ti Plates/Implants with the Modified IGF-1s

Pure Ti samples (included Ti specimens 2 × 32 × 32 mm, Zhong Bang Corporation, Xi’an, China) were divided into four groups: machined-surface group (control), minTBP-1-IGF-1 group, 2minTBP-1-IGF-1 group, and 3minTBP-1-IGF-1 group. Before use, all samples were ultrasonically cleaned with acetone and 70% (*v*/*v*) ethanol for 20 min, followed by sterile deionized water for 10 min. The samples were then dried and sterilized with irradiated cobalt 60. For protein adsorption, first, the modified IGF-1s were at a concentration of 0.1 mg/mL dissolved in binding buffer (5 M urea, 0.2 M Tris–HCl (pH 9.5), 0.1% Tween 20), then samples were placed into 10 mL the protein solution at ambient temperature for 1 h, finally, the samples were washed three times with binding buffer and TBS.

### 2.2. Surface Characterization

Surface morphology and roughness were observed by atomic force microscopy (AFM, Vecco Instrument Dimension, Icon, Aschheim, Germany). Measurements were carried out at scan size of 3 μm and at scan rate of 0.3 Hz.

### 2.3. Water Contact Angle Test

The surface hydrophilicity was assessed by contact angle measurements using Automatic Contact Angle Meter Model SL200B (Solon, Shanghai, China), which was conducted at room temperature by dropping 2 μL of distilled water onto the surface.

### 2.4. Assaying Adsorbed Modified IGF-1s

Modified IGF-1s were quantified using a Human IGF-1 ELISA development kit (PeproTech, Rocky Hill, NJ, USA). To estimate the amount of adsorbed protein, specimens were immersed in the modified IGF-1s solution for 30 min, 1, 6, 24 h at ambient temperature. Afterward, the specimens were put into 5 M urea, 0.2 M HCl, and 0.1% Tween 20 for 30 min [22]. This eluate was diluted 1:20 in PBS containing 0.1% BSA before carrying out ELISAs.

### 2.5. T2DM Animal Model

The design and implementation of the animal experiment were carried out with the permission of the Animal Ethics Committee of the School of Stomatology (Air Force Medical University, Xi’an, China). A total of 18 male Sprague-Dawley (SD) rats (260–300 g) were purchased from the animal holding unit of Air Force Medical University. As previous report, a high-fat diet (consisting of 48% carbohydrate, 22% fat, and 20% protein, with a total calorific value of 44.3 kJ/kg) and low-dose streptozotocin (STZ, Sigma, Neustadt, Germany) intraperitoneal injection were applied to SD rats to induce T2DM [7]. Rats received intraperitoneal injection of streptozotocin with a dose of 30 mg/kg after five weeks of high-fat diet. Four weeks following the STZ injections, rats with blood glucose levels of ≥16.7 mmol/L were used for experiments.

### 2.6. Culture of Cells

Osteoblasts were isolated from the calvaria of neonatal SD rats by enzyme separation method. Briefly, the calvaria tissues were digested after shredding with 0.5% trypsin for 15 min at 37 °C. After being washed intensively with PBS, the segments were transferred to culture flask under normal culture conditions: Dulbecco’s modified Eagle’s medium (DMEM, Gibco) supplemented with 10% fetal calf serum (FCS, Gibco, Waltham, MA, USA), 50 mg/mL gentamicin (Sigma), 100 mg/mL ampicillin (Sigma) at 37 °C in a humidified atmosphere of 5% CO_2_. The cells between passage 2 and passage 4 were used in the following experiments. The cells were incubated with diabetic serum which was acquired from T2DM rats.

### 2.7. Cell Attachment and Proliferation Assay

The Cell Counting Kit-8 (CCK-8, Dojindo, Kumamoto, Japan) assay was used to evaluate the cell attachment and proliferation ability. A 500 μL aliquot of suspended cells at a density of 1 × 10^4^ cells mL^−1^ was seeded on different sample surfaces, then incubated for about 6 h for attachment assay and 1, 3, 6 d for proliferation assay. At these four time points, the culture medium was removed, and an aliquot of CCK-8 (10%, 100 μL) dissolved in media was added to each well, after which the plates were incubated for 2 h. The CCK-8-containing solution was transferred to a 96-well plate, and the absorbance at 450 nm was measured using a spectral scanning multimode reader (Varioskan Flash, Thermo Scientific, Waltham, MA, USA).

### 2.8. Cell Morphology

After culturing for 7 days, the samples were rinsed with PBS, fixed with 2.5% glutaraldehyde for 10 min, dehydrated with gradient alcohol, and sputter-coated with a thin gold layer, finally surveyed by field emission scanning electron microscopy (FE-SEM, JEOL JSM-6460).

### 2.9. Alkaline Phosphatase Activity and Detection of the Mineralized Product

After osteogenic induction for 7 days, osteoblasts were washed twice with PBS, fixed with 4% PFA, and stained with the BCIP/NBT ALP Color Development Kit (Beyotime, Haimen, China) to qualitatively evaluate the ALP activity. In the quantitative assay, the cells were lysed in 0.5 mL of distilled water through four standard freeze-thaw cycles. The lysate mixed with the solution of ALP Assay Kit (Beyotime) was incubated at 37 °C for 30 min. The OD value was detected using a microplate reader at 405 nm. The ALP levels were normalized to the total protein content determined by the bicinchoninic acid (BCA) Protein Assay Kit (Beyotime).

Matrix mineralization of osteoblasts was evaluated by Alizarin Red S (Sigma) staining. After induction for 14 days, the samples were fixed in 75% ethanol for 1 h followed by staining with 40 mM Alizarin Red S (pH 4.2) for 30 min at room temperature. The unabsorbed stain was removed by rinsing with distilled water and dried for the qualitative assay. Quantitative analysis was performed by eluting the adsorbed stain to 500 μL of 10% cetylpyridinium chloride in 10 mM sodium phosphate (pH 7.0) for 2 h and the optical density at 570 nm was measured on the microplate reader.

### 2.10. Osteogenesis-Related Gene Expression

Expression of osteogenesis-related genes was evaluated using quantitative real-time PCR assay. Osteoblasts were seeded with 2 × 10^4^ cells/cm^2^ and cultured for 7 and 14 days. Total RNA was subsequently extracted using TRIzol (Thermo-Fisher Scientific) and first-strand complementary DNA (cDNA) was synthesized using a cDNA synthesis kit (Promega). Quantitative real-time PCR was then performed using rat ALP, BMP-2, OCN, OPG, and Runx2 primers, and Fast SYBR Green Master Mix in a Step One Plus™ Real-Time PCR System (Applied Biosystems, Carlsbad, CA, USA). The primers were designed and synthesized by Genecopoeia (China). The expressions of the genes were normalized to the mRNA levels of the internal control β-actin.

### 2.11. Statistical Analysis

The data were expressed as mean ± standard deviation. The statistical significance of differences was determined by one-way analysis of variance (ANOVA) and Tukey’s multiple comparison tests using SPSS 16.0 statistical software (SPSS, Chicago, IL, USA). The difference was considered to be significant and highly significant when *p* < 0.05 and 0.01, respectively.

## 3. Results

**Figure 2:**
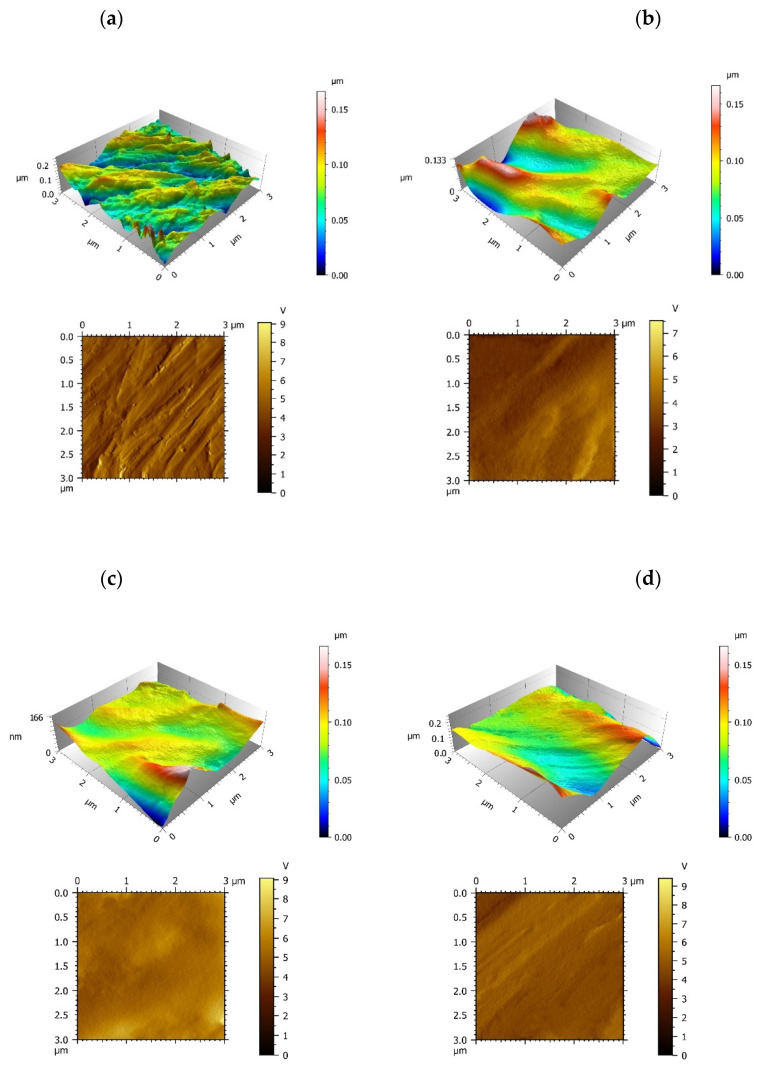

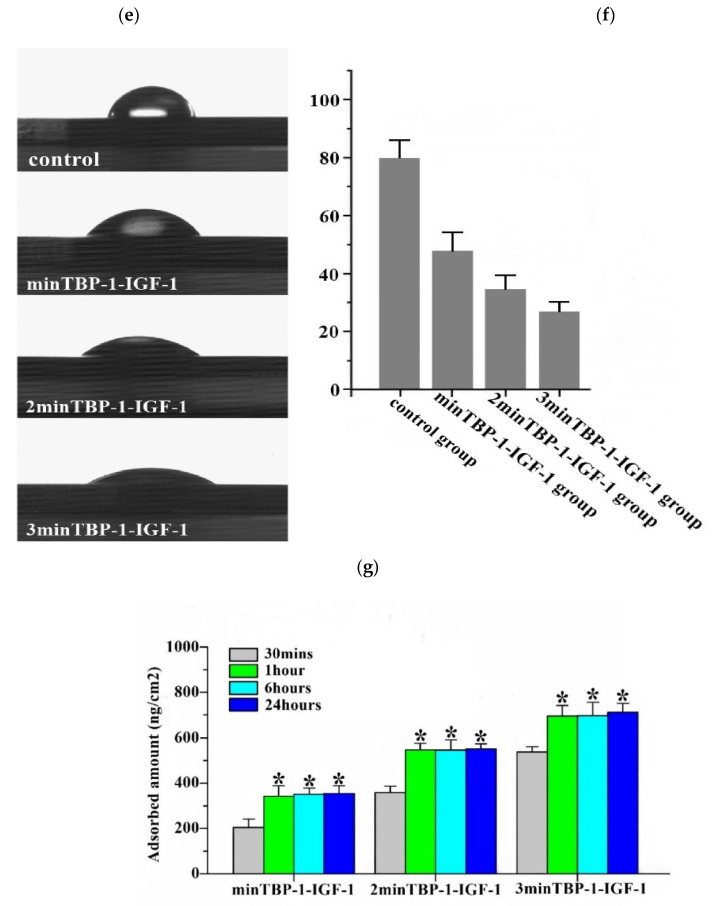
Surface characteristics of each group and adsorption capacity of the modified protein. (**a**–**d**) AFM images of machined surface and (n)minTBP-1-IGF-1-Ti biointerfaces. (**e**,**f**) Water contact angle of the interfaces. (**g**) The amounts of modified IGF-1s adsorbed on Ti surfaces. *n* = 10, * *p* < 0.05.

To study the surface properties, the roughness and topography of the samples interface were analyzed by AFM. The control group exhibited a coarse surface revealing Ra value of 7.59 nm on average, and there are a great deal of bulges and gullies on machined Ti surface (Figure 2a). The three modified IGF-1 groups involved a reduction of the roughness revealing Ra(nm) values of 4.07 (minTBP-1-IGF-1 group), 3.56 (2minTBP-1-IGF-1 group) and 2.83 (3minTBP-1-IGF-1 group) in average, respectively (Table 1). In the minTBP-1-IGF-1 group and the 2minTBP-1-IGF-1 group, proteins were arranged in lumps which indicated that these two kinds of proteins had limited adsorption capacity and could not completely cover the Ti surface (Figure 2b,c). However, in the 3minTBP-1-IGF-1 group, the decrease in Ra value was the greatest, and the 3minTBP-1-IGF-1 protein was distributed evenly on Ti surface as membrane (Figure 2d).

The interface wettability was characterized by the water contact angle measurement. From Figure 2e, the contact angle between droplet and interface decreases gradually from the control group to the 3minTBP-1-IGF-1 group. Water contact angle measurement results (Figure 2f) showed that the surface wettability changes from 79.4 ± 6.6° (control group) to 46.5 ± 6.9° (minTBP-1-IGF-1 group), 34.4 ± 4.7° (2minTBP-1-IGF-1 group), and 28.2 ± 3.1° (3minTBP-1-IGF-1 group). These results demonstrated that the modified IGF-1s promoted surface hydrophilicity (Figure 2e) and the 3minTBP-1-IGF-1-Ti interface showed the strongest hydrophilicity.

The densities of the three modified IGF-1s adsorbed on the Ti surface were 343 ± 39 ng/cm^2^ (minTBP-1-IGF-1), 537 ± 27 ng/cm^2^ (2minTBP-1-IGF-1), and 695 ± 44 ng/cm^2^ (3minTBP-1-IGF-1) separately after one hour of adsorption (Figure 2g), indicating that the affinity between the three kinds of chimeric protein and Ti was different and the adsorption capacity of 3minTBP-1-IGF-1 was the strongest. Figure 2g also verified that the amount of adsorbed modified IGF-1s tended to be stable at 1 h.

Throughout the experiment, the blood glucose level of the SD rats was stable at around 18.6 mmol/L from four weeks after T2DM induction.

**Figure 3:**
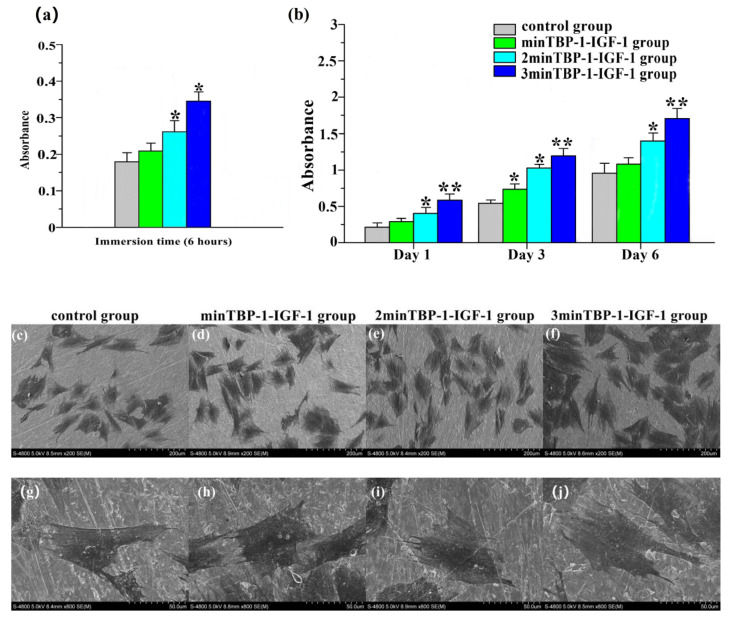
(**a**) Absorbance of osteoblast attachment by CCK-8 assay after 6 h of incubation. (**b**) Absorbance of osteoblast proliferation by CCK-8 after culturing for 1, 3, and 6 days on machined Ti and the (n)minTBP-1-IGF-1 interfaces. (**c**–**f**) SEM images of osteoblasts after culturing for 7 days on different samples at lower magnification (×200). (**g**–**j**) SEM images of osteoblasts after culturing for 7 days on different samples at higher magnification (×800). *n* = 10, * *p* < 0.05, ** *p* < 0.01.

The quantitative results of cell attachment were further corroborated by CCK-8 assay (Figure 3a). The results showed that after culturing for 6 h, more cells were attached to the modified interfaces than to the machined surface. There was no significant difference in absorbance between the control group and the minTBP-1-IGF-1 group (*p* > 0.05). In addition, the absorbance of the 3minTBP-1-IGF-1 group was much higher than those of the other groups.

The CCK-8 assay was employed to evaluate the proliferation level of osteoblasts culturing for 1, 3, and 6 days of different groups (Figure 3b). The control group and the minTBP-1-IGF-1 group demonstrated lower absorbance values when compared with the 2minTBP-1-IGF-1 and the 3minTBP-1-IGF-1 groups on day 1. For longer periods of incubation, this tendency was enhanced. On the third day and sixth day, absorbance values of both the 2minTBP-1-IGF-1 group and the 3minTBP-1-IGF-1 group were significantly increased when compared with the control group, and the minTBP-1-IGF-1 group. Additionally, the absorbance of the 3minTBP-1-IGF-1 group was significantly higher than those of the other groups (*p* < 0.05). These results indicated that introducing 3minTBP-1-IGF-1 to the Ti surface significantly increased cell proliferation.

The SEM images of the morphology of osteoblasts cultured on the different interfaces for 7 days are presented in Figure 3c–j. As shown by the lower magnification images (Figure 3c,d), the osteoblasts were less and expressed fusiform morphology with little filopodia and lamellipodia in the control group and the minTBP-1-IGF-1group. On the contrary, the osteoblasts (Figure 3e,f) were more and expressed polygonal with abundant filopodia and lamellipodia in the 2minTBP-1-IGF-1 group and the 3minTBP-1-IGF-1group. Among the four groups, the performance of the 3minTBP-1-IGF-1 group was the most prominent. The higher-magnification images (Figure 3g–j) disclosed that the osteoblasts of the three modified IGF-1s groups had a larger cell spreading area compared with those on the machined surface. Most importantly, the 3minTBP-1-IGF-1 group had the largest cell extension area.

**Figure 4:**
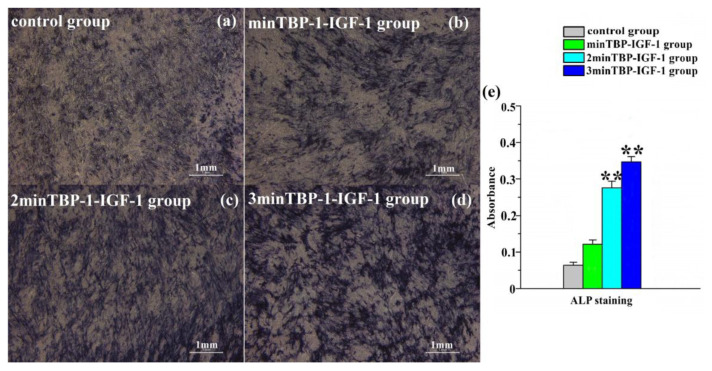

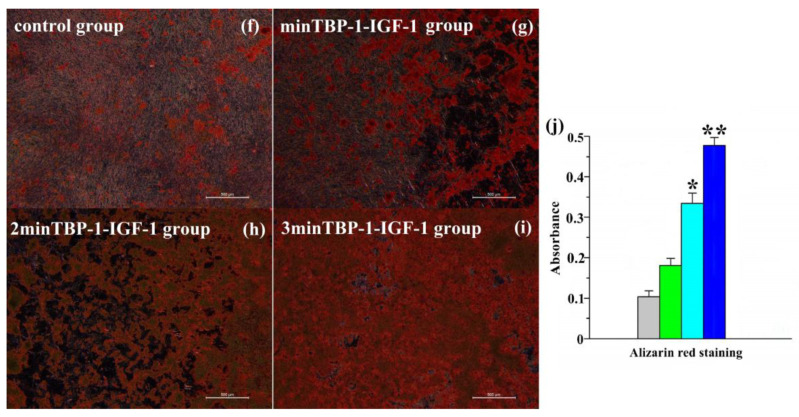
(**a**–**d**) ALP activity was assessed by staining with BCIP/NBT 7d after induction; (**e**) absorbance (405 nm) after ALP staining; (**f**–**i**) images of mineralized clots were obtained by alizarin red S staining 14 d after osteogenesis induction; (**j**) absorbance (570 nm) after alizarin red S staining. *n* = 10, * *p* < 0.05, ** *p* < 0.01.

To determine whether (n)minTBP-1-IGF-1 promoted osteogenic differentiation, we measured the ALP activity and nodule formation of osteoblasts culturing on modified IGF-1s interfaces. The ALP activity results showed that the staining color in modified IGF-1s groups was deeper than that in the control group and the 3minTBP-1-IGF-1 group demonstrated the deepest color after 7d (Figure 4a–d). Furthermore, the absorbance of the 3minTBP-1-IGF-1 group was significantly higher than those of the other groups (Figure 4e) (*p* < 0.05). To evaluate the level of calcification of the nodules secreted by osteoblasts culturing on different interfaces, Alizarin Red Staining was performed at 14d of incubation. The results indicated that secreted bone nodules were dyed red (Figure 4f–i). In the control group and the minTBP-1-IGF-1 group, small size and few numbers of red mineralization nodules were observed. However, 2minTBP-1-IGF-1 and 3minTBP-1-IGF-1 induced more and larger min-eralization nodules, especially the 3minTBP-1-IGF-1 group showed the most uniform red color which demonstrated the best mineralization capacity. The result of Alizarin Red Staining showed (Figure 4j) that the absorbance of the 3minTBP-1-IGF-1 group was significantly higher than those of the other groups (*p* < 0.05).

**Figure 5:**
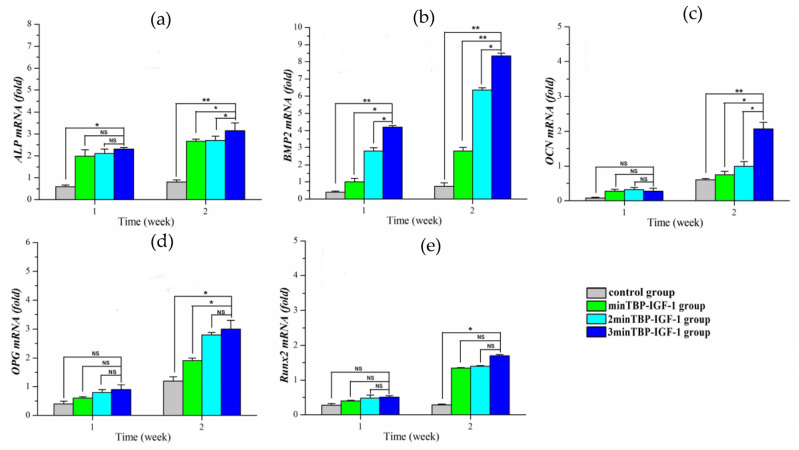
The gene expression of osteogenic markers ((**a**) ALP, (**b**) BMP-2, (**c**) OCN, (**d**) OPG, and (**e**) Runx2) were detected by qRT-PCR. *n* = 10, NS: no statistical difference,* *p* < 0.05, ** *p* < 0.01.

To understand the possible mechanisms by which (n)minTBP-1-IGF-1s stimulated cell mineralization, we investigated their effects on the expression of osteogenesis-specific marker genes, ALP, BMP-2, OCN, OPG, and Runx2 in osteoblasts, and they were normalized to the housekeeping gene β-actin. In general, the expression of osteogenesis-promoting genes increased over time in all groups. In the first week, the expression of ALP and Runx2 (Figure 5a,e) increased significantly in the modified IGF-1 groups compared to the control group. Although there was no difference among the modified IGF-1 groups, the ALP and Runx2 expression of the 3minTBP-1-IGF-1 group was the highest. In the second week, the ALP expression of the 3minTBP-1-IGF-1 group increased the most. Compared with the control group, the expression of Runx2 in the 3minTBP-1-IGF-1 group increased from 1.58 times in the first week to 4 times in the second week. In BMP-2 expression (Figure 5b), there were significant differences between the 3minTBP-1-IGF-1 group and the other groups. In the first week, there was no difference in the expression of OCN and OPG among groups (Figure 5c,d). In the second week, the OCN and OPG expression of the modified IGF-1 groups was significantly higher than that of the control group. Especially the 3minTBP-1-IGF-1 group showed the highest expression level among the three modified IGF-1 groups. These results suggested that 3minTBP-1-IGF-1 can effectively promote osteogenic gene expression.

## 4. Discussion

Due to the relationship between T2DM and periodontitis, a large proportion of patients with tooth loss in the T2DM population. An implant-supported denture is a good way to treat tooth loss and patients with T2DM have great demand for implant therapy. There is increasing evidence that T2DM could affect implant success due to poor bone-implant osseointegration [4]. Given that the implant surface is a key point of successful osseointegration during the early stage of bone healing, numerous studies have focused on enhancing dental implants’ stability by modifying the surface properties of the implants, to minify the failure rate and recovery time after implantation [2,6,7,10,11]. The development of surface-modified implants by increasing micro roughness or changing their chemical composition has been a major research hotspot in recent years [1]. However, there are few studies on different surface modification effects in the DM environment [4]. Osteoblasts play an important role in bone matrix formation and mineral deposition in osseointegration. A large number of studies have confirmed that hyperglycemia has direct and indirect effects on the function and differentiation of osteoblasts, thus affecting bone mass [20,21]. In vitro studies have shown that hyperglycemia directly affects the metabolism and maturation of osteoblasts by changing gene expression, so as to reduce the quality of bone minerals [21,22]. In addition, hyperglycemia has been shown to increase the level of human proinflammatory cytokines and nuclear factors- κb receptor activator expression of ligand (RANKL) [23] mediates osteoblast death and osteoclastogenesis [24].

Lots of experiments have confirmed that IGF-1 plays an important role in improving bone formation and mineralization in T2DM conditions [25]. Additionally, in vitro studies have confirmed that IGF-1 can protect cell function from the negative effects of high glucose levels [25]. Therefore, how to introduce IGF-1 to the implant surface becomes the focus we pay attention to. A previous experiment has confirmed that nucleic acid aptamer minTBP-1 with high affinity to pure titanium can carry DNA polymerase and self-assemble on the Ti surface. In this experiment, we used minTBP-1 as the carrier of IGF-1 to self-assemble on the Ti surface [26].

We prepared three fusion proteins. The AFM images (Figure 2a–d) showed that the more minTBP-1 contained, the more homogeneous protein adsorption on the Ti surface. In addition, the ELISA experiment (Figure 2g) confirmed that the more minTBP-1 contained, the greater adsorbed amount of the fusion protein. Therefore, a sufficient amount of 3minTBP-1-IGF-1 can form a film on the Ti surface, then the roughness was significantly reduced and the hydrophilicity was remarkably improved. Surface wettability plays an important role in the improvement of early bone healing through cell adhesion, protein adsorption, and platelet adhesion [27,28]. Researchers have analyzed the relationship between material surface hydrophilicity and cell biological behavior and found that compared with the hydrophobic surface the hydrophilic surface is more beneficial to cell early adhesion/proliferation [2,27]. In a subsequent experiment, it was confirmed that the surface modified with the 3minTBP-1-IGF-1 could most effectively promote cell adhesion.

Cell attachment, belonging to the first phase of cell-biomaterial interaction, acts as a key role in regulating cell proliferation [29]. The good initial adhesion activity of cells on the biomaterial surface can promote later proliferation and differentiation [30]. The CCK-8 assay demonstrated that the absorbance value of 3minTBP-1-IGF-1group was the highest, which indicated that 3minTBP-1-IGF-1 protein could effectively promote cell attachment in diabetic conditions. After adhering to the substrate, the cells begin to spread. The cytoskeleton composed of microtubules, microfilaments (F-actin), and intermediate filaments [31] not only maintains the cell shape but also participates in cell proliferation and differentiation [30,32]. The SEM results confirmed that osteoblasts on 3min TBP-1-IGF-1 coating spread best, and the cells had already fused after 7 days. The broad cell adhesion and spreading on the 3minTBP-1-IGF-1 modified surface was beneficial for subsequent proliferation and differentiation, considering that the osteoblast differentiation is benefited by good cell spreading [33,34] and cell-to-cell communication [35,36].

After osteoblasts were planted on specimens for 1, 3 and 6 days, the CCK-8 assay confirmed that the absorbance values of the 3minTBP-1-IGF-1 group were always the highest, which indicated that introducing 3minTBP-1-IGF-1 to Ti surface significantly increased cell proliferation level. It is well known that IGF-1 promotes cell proliferation and inhibits cell apoptosis [36,37,38,39]. The above experiments confirmed that the prepared fusion protein can effectively play the role of IGF-1.

Hyperglycaemia has been shown to reduce the rate of bone formation markers including osteocalcin, alkaline phosphatase [5,6,7,8], and procollagen type 1 N-terminal propeptide (PINP). The reduced rate of bone turnover markers together with changes to the organic matrix and cortical structure results in an overall deterioration of the quality, resilience, and structure of the bone tissue [40]. It had been confirmed that IGF-1 plays an important role in regulating bone cell metabolism. Additionally, it stimulates ECM synthesis and affects proliferation, phenotypic gene expressions, and mineralization of osteoblasts [7,41,42]. ALP and alizarin red staining were introduced to detect the early and late osteogenic differentiation of cells, respectively. From Figure 4, both stainings in the 3minTBP-1-IGF-1 group were the deepest, suggesting that 3minTBP-1-IGF-1 can play a short-term and long-term role in improving osteoblasts mineralization.

In vitro, high glucose has been shown to inhibit the osteogenic differentiation/proliferation of osteoblasts and reduce the expression of pro-osteogenic markers such as Runx2 and Osterix. In order to verify the effect of the fusion protein, we detected five osteogenesis-related genes in this study, namely, ALP, OCN, OPG, BMP-2, and Runx2. ALP is an abundant protein expressed in the early stages of osteoblastic differentiation [43], and its expression is prior to that of OCN and OPG [44]. Runx2 binds to the promoter regions of all osteogenesis-specific genes and acts as a master regulator to control the expression of downstream osteoblastic markers during osteoblasts differentiation [45]. Some studies showed that at the early stage of osteoblast differentiation high expression of BMP-2 can activate the expression of ALP and Runx2 downstream [44]. Therefore, in the early stage, the expression of the BMP-2 gene was the earliest and strongest among the five genes. PCR results showed that in the first week, although the expression of ALP and Runx2 in the 3minTBP-1-IGF-1 group was the highest, there was no significant difference between the three experimental groups. OCN was reported to be involved in regulating the formation of mineralization matrix and was recognized as the marker that appeared in the late stage of osteogenic differentiation and as the representative of mature osteoblastic cells [44]. OPG was one of the new biochemical markers for bone metabolism, which can contribute to bone formation in a long steady period [44]. Thus, there was no difference in the expression of OCN and OPG genes among the four groups in the first week. In the second week, the gene expressions in the three experimental groups were significantly different from that in the control group, and the expression in the 3minTBP-1-IGF-1 group was the highest. These findings are consistent with the previous report manifested that IGF-1 promotes the differentiation and maturation of osteoblasts by inducing Runx2 expression with attendant increases in the levels of ALP, OCN, and OPG [45].

The limitations of this study include that in vitro results are not validated in vivo. First of all, the internal environment is very complex, especially in diabetes; secondly, there may be a loss of adsorbed modified IGF-1s during implantation. Therefore, the validation of the (n)minTBP-1-IGF-1-implant complex in vivo is particularly important. We will continue this work in the follow-up experiments

## 5. Conclusions

The fabrication of (n)minTBP-1-IGF-1 on the Ti surface provides a new method for implant surface modification. 3MinTBP-1-IGF-1 showed the strongest affinity with Ti, and the interface demonstrated the strongest surface hydrophilicity and the most homogeneous state. The in vitro biological evaluation results showed that 3minTBP-1-IGF-1 modified surface promoted osteoblasts adhesion, proliferation, spreading, alkaline phosphatase activity, mineralized product, and osteogenesis-related gene expression, superior to the other groups in diabetic conditions. It was confirmed that minTBP-1 could be used as an effective carrier of IGF-1 and the 3minTBP-1-IGF-1 had the most obvious effect on the osteogenic activities. All the above results suggest that 3minTBP-1-IGF-1 is a convenient, effective, and functional protein that enhances osteogenesis on the implant Ti surface. Additionally, this study may provide new strategies for implant repair in T2DM patients.

## Figures and Tables

**Table 1:** Ra values of surface roughness of the four experimental groups.

Group	Ra (nm)
control group	7.59 ± 0.62
minTBP-1-IGF-1 group	4.07 ± 0.41 *
2minTBP-1-IGF-1 group	3.56 ± 0.47 *
3minTBP-1-IGF-1 group	2.83 ± 0.32 **

*n* = 10; * *p* ≤ 0.05; ** *p* ≤ 0.01.

## Data Availability

Data is contained within the article.
